# The Poly-Histidine Tag H6 Mediates Structural and Functional Properties of Disintegrating, Protein-Releasing Inclusion Bodies

**DOI:** 10.3390/pharmaceutics14030602

**Published:** 2022-03-10

**Authors:** Julieta María Sánchez, José Vicente Carratalá, Naroa Serna, Ugutz Unzueta, Verónica Nolan, Alejandro Sánchez-Chardi, Eric Voltà-Durán, Hèctor López-Laguna, Neus Ferrer-Miralles, Antonio Villaverde, Esther Vazquez

**Affiliations:** 1 Institut de Biotecnologia i de Biomedicina, Universitat Autònoma de Barcelona, Plaça Cívica s/n, Bellaterra, 08193 Barcelona, Spain; jsanchezqa@gmail.com (J.M.S.); josevicente.carratala@uab.cat (J.V.C.); srnaroa@gmail.com (N.S.); eric.volta@uab.cat (E.V.-D.); hector.lopez@uab.cat (H.L.-L.); neus.ferrer@uab.cat (N.F.-M.); 2 Departament de Genètica i de Microbiologia, Universitat Autònoma de Barcelona, Plaça Cívica s/n, Bellaterra, 08193 Barcelona, Spain; uunzueta@santpau.cat; 3 CIBER de Bioingeniería, Biomateriales y Nanomedicina (CIBER-BBN), C/ Monforte de Lemos 3-5, 28029 Madrid, Spain; 4 Instituto de Investigaciones Biológicas y Tecnológicas (IIBYT), CONICET-Universidad Nacional de Córdoba, ICTA & Cátedra de Química Biológica, Departamento de Química, FCEFyN, UNC. Av. Velez Sarsfield 1611, Córdoba X 5016GCA, Argentina; vnolan@unc.edu.ar; 5 Biomedical Research Institute Sant Pau (IIB Sant Pau), Sant Antoni Maria Claret 167, 08025 Barcelona, Spain; 6 Josep Carreras Leukaemia Research Institute, 08025 Barcelona, Spain; 7 Servei de Microscòpia, Universitat Autònoma de Barcelona, Plaça Cívica s/n, Bellaterra, 08193 Barcelona, Spain; alejandro.sanchez.chardi@uab.cat; 8 Departament de Biologia Evolutiva, Ecologia i Ciències Ambientals, Facultat de Biologia, Universitat de Barcelona, Av. Diagonal 643, 08028 Barcelona, Spain

**Keywords:** inclusion bodies, functional amyloids, protein secretion, His–cation coordination, biomaterials, protein materials, slow protein release, drug delivery

## Abstract

The coordination between histidine-rich peptides and divalent cations supports the formation of nano- and micro-scale protein biomaterials, including toxic and non-toxic functional amyloids, which can be adapted as drug delivery systems. Among them, inclusion bodies (IBs) formed in recombinant bacteria have shown promise as protein depots for time-sustained protein release. We have demonstrated here that the hexahistidine (H6) tag, fused to recombinant proteins, impacts both on the formation of bacterial IBs and on the conformation of the IB-forming protein, which shows a higher content of cross-beta intermolecular interactions in H6-tagged versions. Additionally, the addition of EDTA during the spontaneous disintegration of isolated IBs largely affects the protein leakage rate, again protein release being stimulated in His-tagged materials. This event depends on the number of His residues but irrespective of the location of the tag in the protein, as it occurs in either C-tagged or N-tagged proteins. The architectonic role of H6 in the formation of bacterial IBs, probably through coordination with divalent cations, offers an easy approach to manipulate protein leakage and to tailor the applicability of this material as a secretory amyloidal depot in different biomedical interfaces. In addition, the findings also offer a model to finely investigate, in a simple set-up, the mechanics of protein release from functional secretory amyloids.

## 1. Introduction

In the context of sustained drug delivery, a diversity of advanced materials for controlled release are under continuous development [1,2,3,4]. Most of them are based on mechanically stable, biologically inert networks or scaffolds that act as holders of the therapeutic molecule and from, which under physiological conditions, a leakage of this payload is allowed or promoted [5,6,7]. This strategy is of particular interest in regenerative medicine that requires the time-prolonged local release of hormones and growth factors [8,9], and in other clinical conditions in which steady systemic drug levels are necessary to enhance efficacy, in contrast to undesired drug oscillations [10,11,12,13,14]. Considering the potential toxicity and limited biocompatibility of the potential scaffolds, self-disintegrating materials in which the drug itself also acts as a mechanically stable network, in absence of vehicle, would be more interesting [15]. Due to the combination of structural and functional traits, protein drugs are especially suitable for this self-containing approach. In this regard, the secretory granules from the human endocrine system are functional amyloids in which peptide hormones are self-stored through ion-assisted protein–protein interactions based on histidine residues [16,17,18,19]. This category of interactions between divalent cations such as Zn^2+^ and histidine residues, common in natural amyloidal structures [20,21,22], are fully reversible and ensure the physiological release of peptide hormones into the bloodstream.

On the other hand, bacterial inclusion bodies (IBs) are insoluble clusters of recombinant proteins spontaneously formed in bacterial cell factories [23], considered as natural mimetics of these endocrine secretory protein granules [24]. This is because, in the cytoplasm of recombinant bacteria, part of the overproduced protein aggregates as mechanically stable sub-micron depots of partially folded polypeptides that are slowly released to the protein quality control system, aiming at either chaperone-assisted refolding or proteolytic degradation [25,26,27,28,29]. By exploiting the secretory properties of such bacterial materials, IBs have been tested as time-prolonged delivery systems for recombinant protein drugs. This strategy has been applied in wound healing [30], for subcutaneous [31] or intranasal delivery of antigens [32,33], or for the local [34] or remote [35] delivery of antitumoral proteins, namely pro-apoptotic factors or tumor-targeted microbial toxins, respectively. In veterinary medicine, they have been proved as excellent immunomodulators and nanostructured vaccines [32,36,37,38,39,40], since minor amounts of bacterial cell wall lipopolysaccharides from the producing bacterial cells, associated to these protein granules, probably have a positive role on the immunoprotection reached [41].

Artificial IB versions have been fabricated in vitro from pure protein to avoid contamination with bacterial molecules, by the engineering of cross-interactions between histidine-rich tags in His-tagged proteins through coordination with divalent cations such as Zn^2+^ or Ca^2+^ [42]. However, the potential role of histidine-rich tags and ions in the in vivo formation of natural IBs in the bacterial cytoplasm has been never explored, despite its obvious interest. If His-rich peptides have a role in the physiological aggregation of recombinant proteins, IB formation and properties may be manipulated, a possibility that would expand the versatility of such natural materials. This is particularly important since His-rich peptides, such as the hexahistidine (H6), are very often used as purification tags of recombinant proteins and, therefore, present in most of the recombinant polypeptides produced in research and industry [43].

## 2. Materials and Methods

### 2.1. Proteins and IB Production

**Figure 1:**
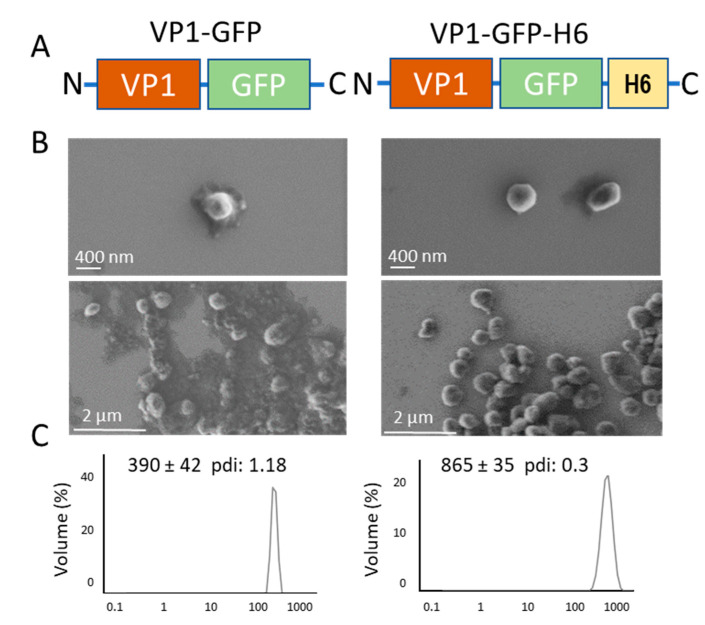
Characterization of bacterial IBs. (**A**). Modular organization of the IB-forming proteins from N- to C- terminal ends. Box sizes are only approximate. (**B**). Representative FESEM images of detail and overview of isolated IBs. (**C**). Hydrodynamic radio of isolated IBs determined by DLS and represented through volume distribution.

VP1-GFP and VP1-GFP-H6 are untagged and H6-tagged versions of a protein fusion between the aggregation-prone viral capsid protein 1 from foot-and-mouth disease virus protein and the enhanced GFP (Figure 1A), respectively. T22-GFP-H6 contains, instead of VP1, a cationic ligand of the CXCR4 receptor, [44,45] namely the peptide T22 (Figure 1A). Further details of these constructions are given elsewhere [46,47,48]. In addition, several variants of T22-GFP-H6 were included as appropriate controls for some experiments. They were the H6-lacking T22-GFP [49], H6-GFP-T22, interchanging N- and C-terminal domains with respect to the parental protein [50], and T22-GFP-H3A and T22-GFP-H5T, in which the number of His residues in the C-terminal tag was reduced from 6 to 3 and 5, respectively [51]. IB versions of these modular proteins were produced in *E. coli* from encoding plasmid vectors. The expression systems were pTrc99a in MC4100 for VP1-GFP, [47] pETDUET in ClearColi^®^ BL21 DE3 (Lucigen, Madison, WI, USA) for VP1-GFP-H6 and pET22b (Novagen, Plédran, France) in Origami B for T22-GFP-H6 and derived constructs [52]. For production of IBs, bacterial cells were cultured in 2 L shake flasks in 600 mL of Lysogenic Broth (LB). Recombinant gene expression was triggered by 1 mM isopropyl β-D-1-thiogalactopyranoside (IPTG) when the OD_550_ reached around 0.5 units. Cells were kept for 3 h at 37 °C in agitation at 250 rpm, and sedimented by centrifugation at 6000× *g* for 15 min at 4 °C. Cell disruption was reached by combined mechanical and enzymatic actions. For that, pelleted cells were resuspended in lysis buffer (50 mM Tris- HCl, 10 mM NaCl, 1 mM EDTA, pH 8). Protease inhibitors, namely phenylmethylsulfonyl fluoride (PMSF) 0.4 mM and cOmplete EDTA-Free (Hoffman Roche, Basel, Switzerland), were also added to minimize proteolysis of the material. Then, 1 μg/mL lysozyme was incorporated, and the mixture was incubated for 2 h at 37 °C, keeping agitation at 250 rpm. Then, three rounds of French Press at 1200 psi were applied, and samples were kept at −80 °C. The cell extracts were thawed, 0.2% *v*/*v* Triton X-100 added, and the mixture incubated for 1 h at room temperature. IBs were recovered and collected by centrifugation at 15,000× *g* for 15 min at 4 °C, and they were finally resuspended in lysis buffer for a final wash. Finally, 0.6 μg/mL DNAse was added and incubated for 1 h at 37 °C. The potential presence of remaining viable bacterial cells was tested by plating IB samples on LB plates. If necessary, samples were submitted to further freeze and thaw cycles until complete absence of colonies on the plates was confirmed. Finally, IBs were washed with deionized sterile water and stored at −80 °C. IB proteins were quantified by Western blotting (WB) using an anti GFP antibody. Known amounts of soluble T22-GFP-H6 were used to generate a standard quantification curve. Using this guide, the amounts of recombinant protein in IBs were estimated by ImageLab software (BioRad, CA, USA), from triplicate blots resulting from independent experiments.

### 2.2. Field Emission Scanning Electron Microscopy (FESEM)

IB geometry and surfaces were studied in a nearly native state with a Field Emission Scanning Electron Microscope (FESEM). IBs in deionized water were deposited over silicon wafers (Ted Pella, Reading, CA, USA) in microdrops of 10 μL. After air drying, uncoated samples were observed in a FESEM Zeiss Merlin (Zeiss, Oberkochen, Germany). The equipment was operated at 2 kV with a high resolution in-lens secondary electron detector [35].

### 2.3. Dynamic Light Scattering

Dynamic light scattering (DLS) was used to determine the volume size distribution of the different IBs. Measurements were carried out in ten replicates for each IB at 25 °C in a Zetasizer Advanced Pro Blue (Malvern Instruments Limited, Malvern, Worcestershire, UK) at 633 nm.

### 2.4. Fourier Transform Infrared Spectroscopy-Attenuated Total Reflectance (FT-IR ATR)

Appropriate IB samples were placed and dried with a continuous N_2_ flow, on spectroscopic crystal surfaces. Total reflectance spectroscopy was determined 16 times in form of spectra. A scan rate of 50 cm^−1^/min with a nominal resolution of 2 cm^−1^ was used, in a Tensor 27 Bruker spectrometer with a Specac’s Golden Gate Attenuated Total Reflectance (ATR) accessory. Measurements were always conducted at 25 °C under a stream of N_2_. The absorbance values were corrected by subtracting the background. Fourier deconvolution of the spectra and the second derivative allowed the identification and analyses of different band components. According to the previously described procedure, fitting the components to the original (not deconvolved) spectrum was performed [53], assuming a Gaussian shape. Data were treated by using the Peakfit software.

### 2.5. Protein Release from IBs

Stored IBs were thawed at RT for several minutes and softly resuspended in 0.1 mL of the corresponding buffer (at 300 µg/mL). Then, 0.1 mL containing different amounts of EDTA was added to achieve a concentration range between 0 and 70 mM. Samples were placed at 37 °C without agitation and after 48 h, the soluble and insoluble fractions were separated by centrifugation at 15,000× *g* for 15 min at 4 °C. The mobility and integrity of the released protein were assessed by SDS-PAGE using a TGX Stain-Free TM FastCastTM Acrylamide Kit, 12% (BioRad), and by Western Blotting (WB). Samples from the soluble fraction were heated at 90 °C for 10 min (and those from the insoluble fraction) for 50 min. Then, they were loaded on SDS-PAGE gels and analyzed by WB with an anti-GFP monoclonal antibody GFP (B-2) sc-9996 Antibody, (Santa Cruz Biotechnology, Inc., Dallas, TX, USA). Images were obtained with the ChemiDoc™ Touch Imaging System (BioRad) and further analyzed with Image Lab Software. The volume band intensity values, obtained from images of blotting membranes, were applied to calculate the percentage of released protein (the fraction of the soluble form in relation to the sum of the soluble and insoluble forms in the IB samples). Image Lab software was used to determine band intensity. When necessary, the ratio of released protein (in %) comparing EDTA-treated samples and the control without EDTA was calculated.

The kinetics of protein release were also analyzed. Samples of 10 mL, containing 3 mg of T22-GFP-H6 IBs, were maintained at 37 °C in the absence or in the presence of 53 mM EDTA. At the desired times, aliquots of 0.1 mL were removed and centrifuged at 15,000× *g* for 15 min at 4 °C. The released protein was determined as described above.

### 2.6. Statistical Analysis

Pairwise data comparisons were analyzed with Student’s t tests. At least three replicates were performed in each experiment. Differences between groups were considered significant at *p* < 0.1. Quantitative values were expressed as the mean ± standard error of the mean (*x* ± SEM). All statistical analyses were performed using Excel version 2016. All the sets of raw data can be found in the Supplementary information.

## 3. Results

To explore the potential role of H6 in the formation of bacterial IBs we produced in Escherichia coli the model protein VP1-GFP, as H6-tagged (VP1-GFP-H6) and untagged forms (Figure 1A). Due to the hydrophobic character of VP1, the major capsid protein of the foot-and-mouth disease virus, modular fusions based on this protein tend to aggregate as IBs upon recombinant production [48]. Both VP1-GFP and VP1-GFP-H6 IBs were mechanically stable, showing a pseudospherical form (Figure 1B), and were distinguishable regarding their reached size. While the average hydrodynamic size of VP1-GFP IBs was around 400 nm, VP1-GFP-H6 IBs peaked at more than 800 nm (Figure 1C).

**Figure 2:**
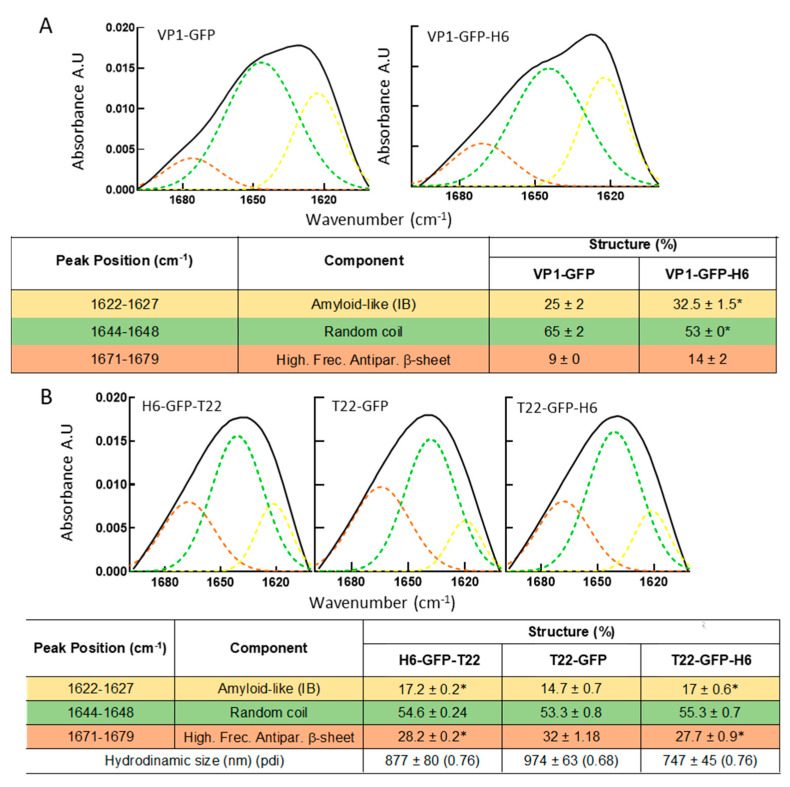
ATR-FTIR spectra of IBs. Amide I band (black) and components resulting from deconvolution (in colors), for two sets of proteins, namely (**A**,**B**). At the bottom, deconvolution results of the spectra analyses as percentages of the components within each IBs. Colored peak components match with the table rows. * Statistically different with respect to the non-tagged equivalent proteins (*p* < 0.1). The VP1-GFP conformational features were in accordance with previous studies applying the same procedure [58].

The conformational quality of VP1-GFP and VP1-GFP-H6 proteins within the respective IBs was evaluated over isolated material by FTIR, a methodology proven of great utility for the study of the IB protein structure [54,55,56]. As observed (Figure 2A), the presence of the H6 tag in VP1-GFP-H6 increased the cross beta intermolecular structure at expenses of random coil protein content that was minimized, the beta intermolecular IB structure being recognized as amyloid-like content [55,56]. An additional IB-forming H6-tagged protein, namely T22-GFP-H6, was incorporated to explore the generic nature of the above observation. In this modular protein, VP1 was replaced by a short cationic peptide (T22), and the modular construct also aggregated in bacteria upon production [57]. Two related proteins, namely the untagged T22-GFP and H6-GFP-T22, were also added as references. As noted (Figure 2B), the relative amounts of amyloid-like and random coil in T22-GFP-H6 and in H6-GFP-T22 were more similar to those of VP1-GFP-H6 IBs than those in VP1-GFP-based materials (Figure 2B). Additionally, the structural profile of VP1-GFP and T22-GFP were similar (Figure 2A,B). These data were indicative of a positive influence of H6 regarding formation of amyloidal structures. This fact could also account for the larger size of VP1-GFP-H6 IBs compared to those formed by the untagged version (Figure 1C) and support the significant contribution of H6 in the protein–protein contacts favoring aggregation in H6-containing fusions.

**Figure 3:**
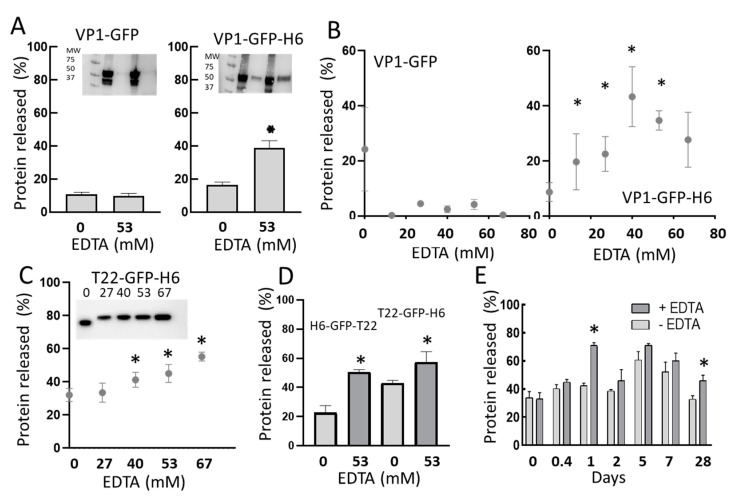
Protein release from IBs. (**A**) Fraction of protein released from untagged or H6-tagged IBs, determined by densitometric analyses of Western Blotting (inset), after 48 h of incubation at 37 °C in buffer or in buffer in presence of 53 mM EDTA. (**B**) Protein release from VP1-GFP and VP1-GFP-H6 IBs under increasing concentrations of EDTA. (**C**) Protein release from T22-GFP-H6 IBs under increasing concentrations of EDTA. Numbers above the Western blot (inset) indicate EDTA concentration in mM. (**D**) Protein release from H6-GFP-T22 and T22-GFP-H6 IBs, in the absence or in the presence of EDTA. (**E**) Time prolonged protein release, with or without EDTA (53 mM), from T22-GFP-H6 IBs. * Statistically different to the condition without EDTA (*p* < 0.1) in all panels.

If, as presumed, H6 has a positive role in the physiological aggregation of recombinant H6-tagged proteins as bacterial IBs, it would be highly plausible that such influence may be exerted via divalent cation coordination, as happens with other natural amyloids [17,20,59,60,61]. As IB proteins are steadily released from the bulk material during its spontaneous disintegration in vivo [35] and in physiological buffers [57], we hypothesized that ion chelation via EDTA would have a differential impact on protein release from bacterial IBs, when comparing H6-tagged with untagged IB proteins. Then, we observed spontaneous protein leakage from both types of IBs 48 h upon incubation with PBS (Figure 3A). In this experimental context, 53 mM EDTA stimulated the release of VP1-GFP-H6 from IBs, while it had no significant effect on the release of VP1-GFP (Figure 3A). Such chemical stimulation was dose-dependent (Figure 3B), as also observed and confirmed in a different model protein, namely T22-GFP-H6, and rather progressive at least until 67 mM EDTA (Figure 3C). Importantly, EDTA-mediated protein release occurred in His-tagged proteins irrespective of the location of the tag. As observed (Figure 3D), the release of the related H6-GFP-T22 and T22-GFP-H6 from their respective IBs was, in both cases, stimulated by chelation mediated by EDTA, a process that was observed during a significant period of time (Figure 3E).

**Figure 4:**
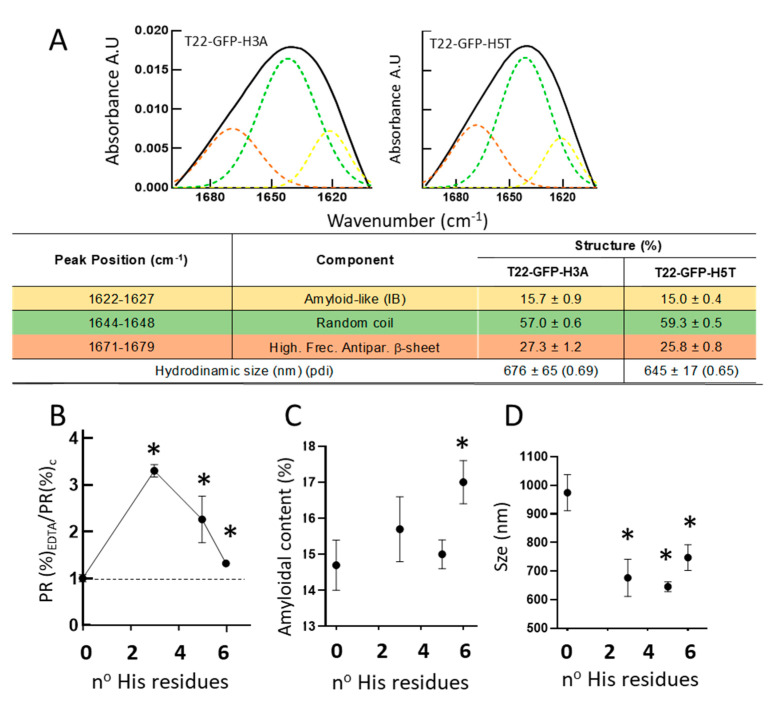
Structural impact of the His residue number on IB properties. (**A**) Amide I band (black) and components resulting from deconvolution (in colors), for two alternative proteins. At the bottom, deconvolution results of the spectra analyses as percentages of the components within each IB. Colored peak components match with table rows. (**B**) Protein release from IBs in the presence of EDTA (53 mM) relative to protein release in the absence of EDTA, represented versus the number of histidine residues in the C-terminal tag. IBs were formed by untagged T22-GFP, by T22-GFP-H6 or by their derivatives with 3 and 5 His residues at the C-terminus. (**C**) Amyloidal content versus His residue number in the tag. (**D**) IB size versus His residue number in the tag. * Statistically different respect to the non-tagged T22-GFP (*p* < 0.1).

To evaluate how the number of His residues in the tag might impact on the EDTA-mediated leakage of IB protein, we compared the ratio of protein release with and without EDTA in a set of closely related, differently tagged proteins. For that, apart from the parental T22-GFP-H6 (6 His residues), we included the untagged T22-GFP (0 His residues), and two T22-GFP-H6 derivatives with three or five His residues in the C-terminal tag, namely T22-GFP-H3A and T22-GFP-H5T, respectively. These two new proteins formed IBs (not shown) and were first analyzed by FTIR (Figure 4A). In all the tested IBs but not in those formed by T22-GFP (as it happened with VP1-GFP, Figure 3A), EDTA stimulated IB protein leakage (Figure 4B). This fact demonstrates that even three clustered His residues in the tag are sufficient for ion-assisted IB formation in vivo. Interestingly, the EDTA-mediated protein release appeared as optimal at this number of His residues, declining when the tag had five or six His residues (Figure 4B). On the other hand, and as inferred from the structural analyses in Figure 2A and Figure 4A, the number of His residues in the tag were directly and positively influencing the amyloid content of the material (Figure 4C). Additionally, for the GFP-based constructs, the presence of the His-based tag reduced the size of the materials from almost 1000 nm to around 700–800 nm, in a way that do not appear to be directly influenced by the number of His residues (Figure 4D).

## 4. Discussion

The present study demonstrates that the addition of an H6 tag to a model fusion protein clearly influences the size of aggregates formed in recombinant bacteria, although this impact is dissimilar depending on the tagged protein (Figure 1B,C and Figure 4D). Probably, the convergence of several factors driving the aggregation process, in which H6 participates, defines the final geometry of this type of protein clusters. Additionally, the presence of the tag enhances the amyloid content of isolated IBs when compared to tag-less equivalent proteins (Figure 2 and Figure 4), and this fact appears to be influenced by the number of His residues in the added peptide (Figure 4C). On the other hand, in vitro chelation of isolated IBs by addition of EDTA enhances, in a dose-dependent manner, the leakage of H6-tagged protein but not of tag-less counterparts (observed in two different models proteins), from respective IBs (Figure 3 and Figure 4B), during the disassembly of the material. Both observations, in combination, support the hypothesis of bacterial IBs being formed, in the bacterial cytoplasm, not only by the mere interaction of homologous hydrophobic protein patches [62,63] but also favored by the cross-molecular interactions between His-rich domains. This would be mediated by chelatable ions recruited from the environment when such clustered His residues are present in the protein. Then, in His-tagged proteins, an at least the dual but, in any case, the multiple category of protein interactions supporting IB formation would then coexist. The optimal value of three His residues, observed in T22-GFP-H3A, indicates an ideal multivalence in each protein building block, within the IBs, that would suit better a chelation-associated disintegration. In other words, three cross-molecular contacts per polypeptide are problably the minimum to allow ion-asssistance in the in vivo IB formation. The ion-assisted IB-formation process appeared to be irrespective of the position of the His-rich tag in the protein, as it was observed in both C-terminal and N-terminal versions of H6-tagged proteins (Figure 3E).

The capability of divalent cations to mediate protein–protein interactions through His-rich domains has been proved in a very different set of contexts [21], including the in vivo formation of amyloidal structures [17,20,59,61,64,65,66,67]. In vitro, H6 and other related peptides have proved useful for the controlled assembly of pure proteins into a set of amyloidal structures with increasing complexity, from nano- to microscales and with clinical applicability [42,49,68,69,70,71,72]. The role of polyhistidines in protein aggregation as bacterial IBs had not been so far explored, even those materials are promising as unconventional drug delivery systems [23,24]. Although the concepts proposed here need further investigation, especially the impact of the tag over the IB size, the presence or absence of His-containing tags in the recombinant protein of interest influences the geometry of the IB material, the conformation of the forming protein and the leakage of the protein by chelation. This is of course critical in aiming to control the secretion rate from IBs for use in biological interfaces. Note that, for recombinant proteins intended to be produced as IBs as a final self-packed form [73], affinity purification mediated by Ni^2+^ chromatography is not required, so the decision to include or not an H6 tag would become merely strategical and based on the intended manipulability and applications of the product.

Apart from the regulation of IB disintegration and protein leakage offered by His-rich peptides to secretion-oriented IBs, these materials represent a convenient model to study the mechanics of protein release from His/metal-based complexes, such as secretory granules from the endocrine system [17,18,19]. Under the need of regulatable protein depots for controlled protein delivery, IBs offer a simple way to understand regulation of protein secretion by relatively simple analytical tools.

## 5. Conclusions

The data presented here strongly support the concept that His-tagging of recombinant proteins strongly impact on the structural architecture of the resulting IBs formed in bacteria, and also on the capability of these materials to sustain the time-prolonged release of functional proteins under physiological conditions. EDTA chelation data also suggest that divalent cations might play a critical role in the scaffolding function of His-rich tags. Since bacterial IBs have been proved clinically relevant as topographies in regenerative medicine, as vaccines and as drug release systems in protein-based therapies, their manipulation through His-tag engineering offers an expanded functional plasticity that might support a refined tailoring of these materials for specific applications.

## Data Availability

Raw data can be found in the Supplementary information and in the permanent repository https://ddd.uab.cat/record/249866?ln=ca (accessed on 4 March 2022).

## Supplementary Materials

The following supporting information can be downloaded at: https://www.mdpi.com/article/10.3390/pharmaceutics14030602/s1.
